# Method matters: Experimental evidence for shorter avian sperm in faecal compared to abdominal massage samples

**DOI:** 10.1371/journal.pone.0182853

**Published:** 2017-08-16

**Authors:** Antje Girndt, Glenn Cockburn, Alfredo Sánchez-Tójar, Hanne Løvlie, Julia Schroeder

**Affiliations:** 1 Evolutionary Biology, Max Planck Institute for Ornithology, Seewiesen, Germany; 2 Department of Life Sciences, Imperial College London, Silwood Park Campus, Ascot, United Kingdom; 3 International Max-Planck Research School (IMPRS) for Organismal Biology, University of Konstanz, Konstanz, Germany; 4 IFM Biology, Linköping University, Linköping, Sweden; Justus-Liebeig University Giessen, GERMANY

## Abstract

Birds are model organisms in sperm biology. Previous work in zebra finches, suggested that sperm sampled from males' faeces and ejaculates do not differ in size. Here, we tested this assumption in a captive population of house sparrows, *Passer domesticus*. We compared sperm length in samples from three collection techniques: female dummy, faecal and abdominal massage samples. We found that sperm were significantly shorter in faecal than abdominal massage samples, which was explained by shorter heads and midpieces, but not flagella. This result might indicate that faecal sampled sperm could be less mature than sperm collected by abdominal massage. The female dummy method resulted in an insufficient number of experimental ejaculates because most males ignored it. In light of these results, we recommend using abdominal massage as a preferred method for avian sperm sampling. Where avian sperm cannot be collected by abdominal massage alone, we advise controlling for sperm sampling protocol statistically.

## Introduction

Male competition over access to females, and sperm competition over fertilisation of eggs, are two sides of the same coin − both determine male reproductive success and ultimately fitness [1,2]. In sexually reproducing species, males compete with each other for access to mates, and when a male fails to secure exclusive copulation rights, his sperm need to outcompete rivals' sperm in fertilising eggs [3]. Sperm competition is ubiquitous across taxa and an important part of sexual selection [2,4]. Thus, one eminent interest of evolutionary biologists is to understand which traits predict the competitiveness of sperm and thus the likeliness to win the sperm race.

In sperm evolutionary ecology research, sperm size and shape matters. Sperm length commonly correlates positively with sperm swimming speed [5–7], but see [8], comparative sperm morphometry (i.e. measured dimensions of different sperm components) is used to reveal phylogenetic relationships and predict sperm energetics [9,10], and variation in sperm morphometry can be indicative of the intensity of sperm competition within species [11–13]. In birds, sperm competition is widespread because of frequent extra-pair copulations in socially monogamous species, polyandrous mating systems or rapid mate switching [14].

Avian sperm biologists have successfully adopted semen collection techniques from the poultry industry [15] and thus can sample sperm from birds with relative ease [16]. Techniques that minimise handling stress and are applicable in the field are desirable because non-domestic birds are often of conservation concern and cannot be kept in captivity. Avian faecal sperm sampling is advocated as a simple and non-invasive alternative to other methods of sperm collection [17]. This technique uses the pathway of passively lost sperm during defaecation to obtain sperm in reproductively active males [18]. An intial study on ten zebra finches, *Taeniopygia guttata*, comparing sperm from faeces and sperm ejaculated into a stuffed female dummy, showed no morphological difference between sperm from both collection techniques [17]. Consequently, sperm collection techniques, such as faecal sperm sampling, dissection of seminal glomera or testes of sacrificed or road-killed birds, female dummy techniques or abdominal massage sperm sampling [19,20] (hereafter called massage), are used interchangeably (e.g. [21–26]). Furthermore, only few studies included the different methods used for sperm sampling in their statistical analysis [27–29] and we are aware of only one [27] that gave detailed information on its effects. For instance, one study has used three methods of sperm collection in the past, accounted for variation in the sampling method statistically by adding collection technique as a random effect, but did not report these estimates [28]. Yet, there is reason to consider that sampling method may sample sperm at different maturational stages [30,31] because intra-testicular sperm, for instance, are less developed than extra-testicular sperm [32–34]. It is currently unclear whether this affects sperm morphometry of sperm sampled with different methods, and if so how one should account for it.

Here, we tested the hypothesis that sperm morphometry does not differ between three sampling methods: samples from males ejaculating into a stuffed female dummy, faecal collection, and massage technique. We used a captive population of house sparrows to repeatedly sample individual males with a randomised design. We measured the length of a sperm's main components: head, i.e. nucleus and acrosome, midpiece and flagellum, and predicted that sampling method does not influence sperm length. In contrast to our prediction, we demonstrate differences in sperm length between the faecal and the abdominal massage method, so mixing sperm sampling methods should be avoided or controlled for statistically to reduce uncertainty in statistical analyses [35,36]. The female dummy did not result in sufficient experimental ejaculates thus statistical analyses of sperm length differences were only conducted between faecal and abdominal massage samples.

## Material and methods

### Study population

Male house sparrows (*n* = 52) were kept at the Max Planck Institute for Ornithology in Seewiesen, Germany, in June 2015. The males were housed in four single-sex semi-outdoor aviaries, single aviary dimensions: 1.2 m x 4.0 m x 2.2 m high, and each aviary contained 13 males. Adjacent walls were covered with hessian fabric to prevent visual contact. The population consists of wild-caught birds in 2005 and 2006 [37] and their offspring. Males were in acoustic, but not visual or physical contact with females for a period of two months before sampling sperm and thus could not copulate with females. Mating can affect sperm depletion and post-meiotic sperm senescence [38,39], which, in our case, can be considered standardised. All individuals were fitted with a numbered metal-ring and a unique combination of three coloured plastic rings for individual identification. Because aviaries had meshed outside walls, light, temperature, humidity and ventilation were close to natural conditions. Additionally, an artificial light-dark cycle was set from 05:30 to 18:00 with light intensity gradually increasing in the morning and dimming in the evening over a period of one hour. Birds were provided with *ad libitum* water and food (wild seed mixture, fresh salad, sunflower seeds, crushed corn and wheat, oats) and mineral mix at all times, as well as sand and water baths. The Government of Upper Bavaria approved the care, handling and husbandry of all birds in this study (Nr 311.5–5682.1/1-2014-024).

### Sperm collection

Sperm were obtained with three different methods: (a) collected with a stuffed female dummy, (b) through faecal collection, and (c) from massage.

#### a) Stuffed female dummy

**Fig 1:**
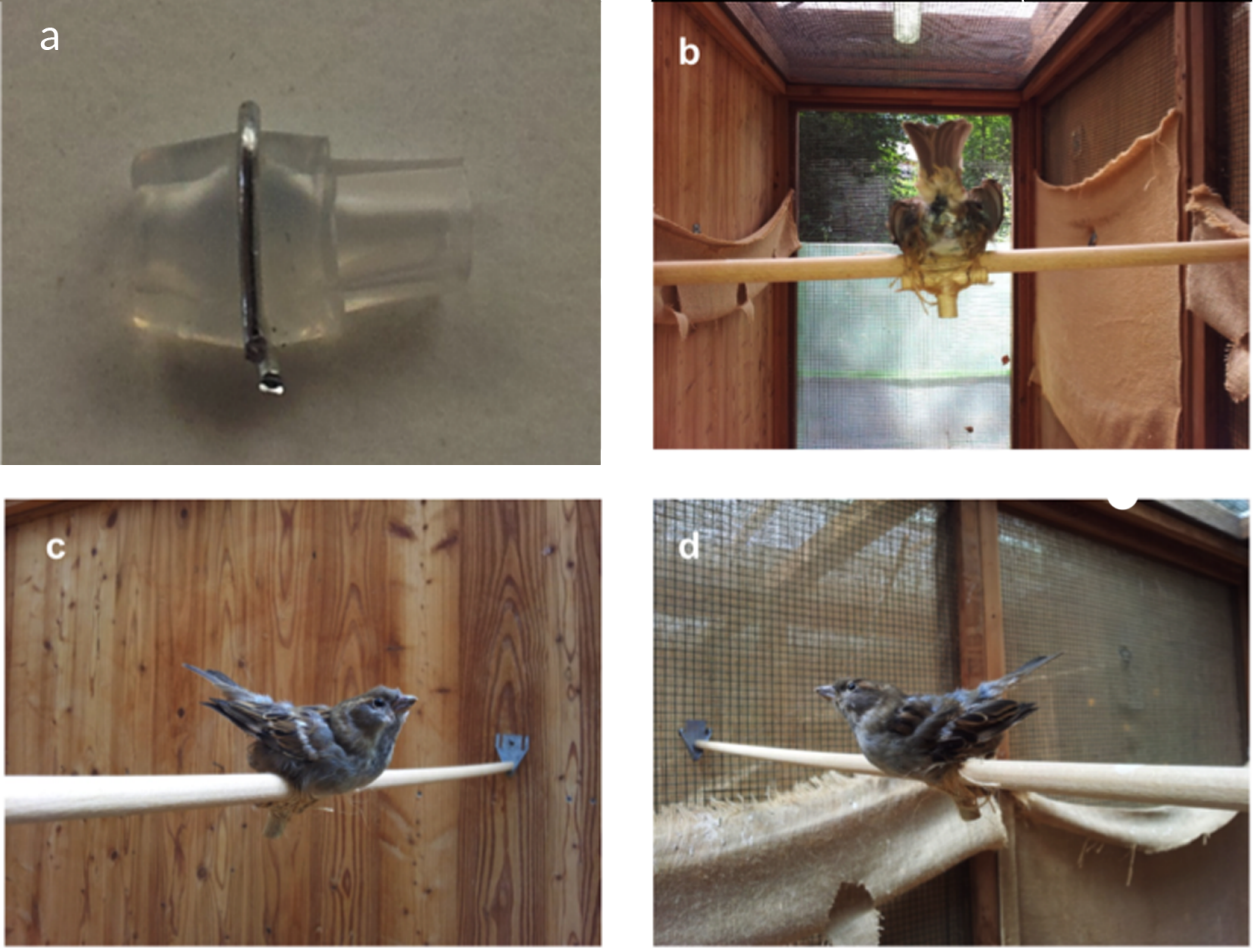
False cloaca and female dummy used for experimental ejaculate collection in male house sparrows. (a) Example of a false cloaca prototype, which was filled at the larger opening with 4μl PBS before being inserted up until the wire into the female dummy. Rear (b), and side (c, d) view of the single female dummy used in sperm collection trials. Pictures courtesy of: Elena Beirer.

The body of one adult female house sparrow that in 2014 had died of a natural cause in our population was skinned, moulded, set-up in copulatory position and fitted with a false cloaca (Fig 1). Cloacae were handmade using medical silicone tubing (inner diameter 1.98 mm) and metal wire (strength 0.7 mm) and filled with 4μl phosphate-buffered saline (PBS) following the design and procedure described in [40] (Fig 1). The false cloaca was then inserted into the female dummy, which was attached to a perch inside the males' aviary and left for a trial period of ten minutes. When a copulation occurred (see S1 Movie in the supporting information), the copulating male was identified by his colour rings, and the experimental ejaculate pipetted from the false cloaca into 200μl of 5% formalin (following [17,40]) for subsequent sperm measurements. Afterwards, the female dummy was equipped with a fresh false cloaca and introduced into the aviary allowing for new copulations.

#### b) Faeces collection

Sperm are continuously released during defaecation in reproductively active males [18] and can be obtained by pipetting any fluid part from fresh faeces as described in [17]. To sample sperm from males' faeces, individual males were removed from their aviary and placed individually inside a cage measuring 60 cm x 40 cm x 45 cm with non-absorbent flooring for a period of ten minutes. Once defaecation occurred, the fluid parts of a male's faeces were pipetted into 200μl of 5% formalin [17].

#### c) Massage

**Fig 2:**
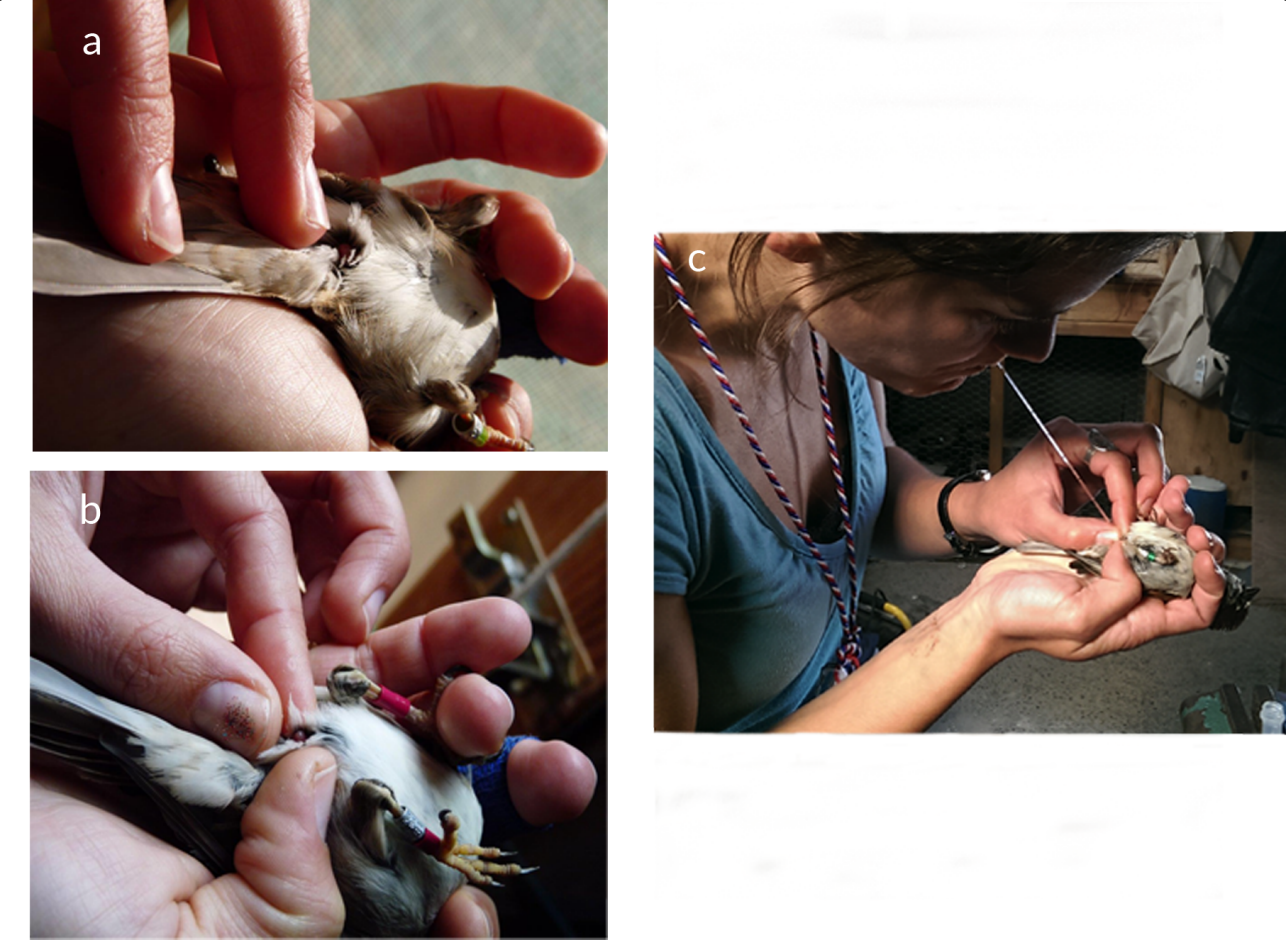
Massage technique used for male house sparrows. (a) A male was positioned on his back and his cloacal protuberance exposed. (b) Pressure was gently applied at the base of the cloacal protuberance using three fingers. (c) Experimental ejaculates were collected using a ring-marked capillary. Pictures a) and b) courtesy of Elena Beirer and c) Julia Schroeder.

In reproductively active male passerines, growth of the seminal glomera results in a swelling called cloacal protuberance, whose main function seems to be sperm storage and maturation [41,42]. Sperm were collected by gently squeezing the cloacal protuberance (Fig 2), which resulted in immediate ejaculation. Individual samples were collected with a 5μl ring-marked capillary (Fig 2) and stored in 200μl of 5% formalin [16,17].

### Experimental protocol for sperm collection

**Fig 3:**
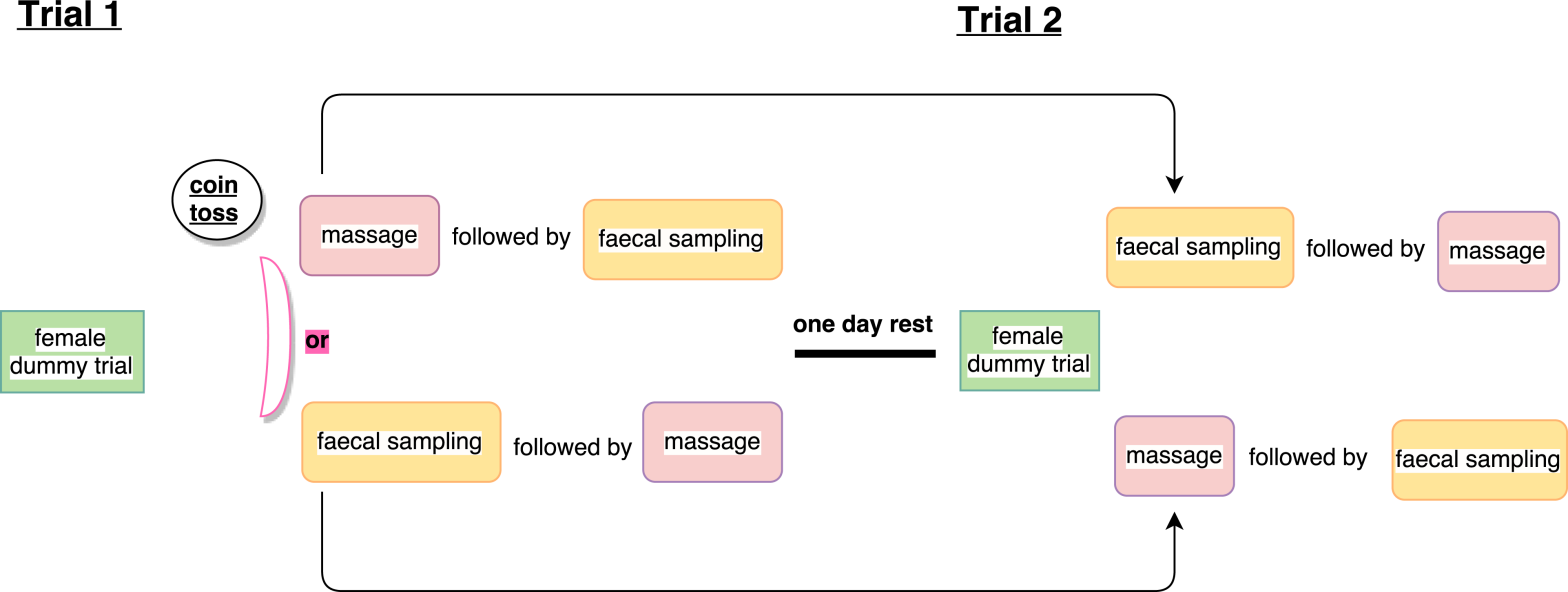
Schematic overview of experimental protocol. Whereas the time and sequence of massage and faecal sampling was randomised, female dummy trials always took place before the faecal and massage sampling.

Sperm sampling took place over four days in mid June, which represented the middle of the house sparrow breeding season [43]. The female dummy collection technique was always performed before faecal and massage sampling because we anticipated that males would be less interested in the female dummy after handling stress from catching. For this purpose, the female dummy was placed inside an aviary for males to copulate with for a period of ten minutes. We proceeded with sperm sampling using the other two methods only after the female dummy test was finished. Therefore, we tossed a coin to randomise whether a male was first sampled by massage, followed by a defaecation trial, or first received a defaecation trial followed by a massage. The whole procedure − first offering the female dummy and then randomising faecal and massage sampling − was repeated for each male after a day's rest. In other words, males were caught again two days after their first trial and depending on their treatment during the first trial, received the reversed sampling order during their second trial. Hence, we aimed to obtain a total of six samples per male: one female dummy, one faecal and one massage sample per day, per male. This protocol randomised massage and faecal sampling in time and sequence to experimentally control for potential order effects on sperm morphometry between methods within sampling days. A schematic overview of the experimental procedure is presented in Fig 3.

### Sperm morphometrics

**Fig 4:**
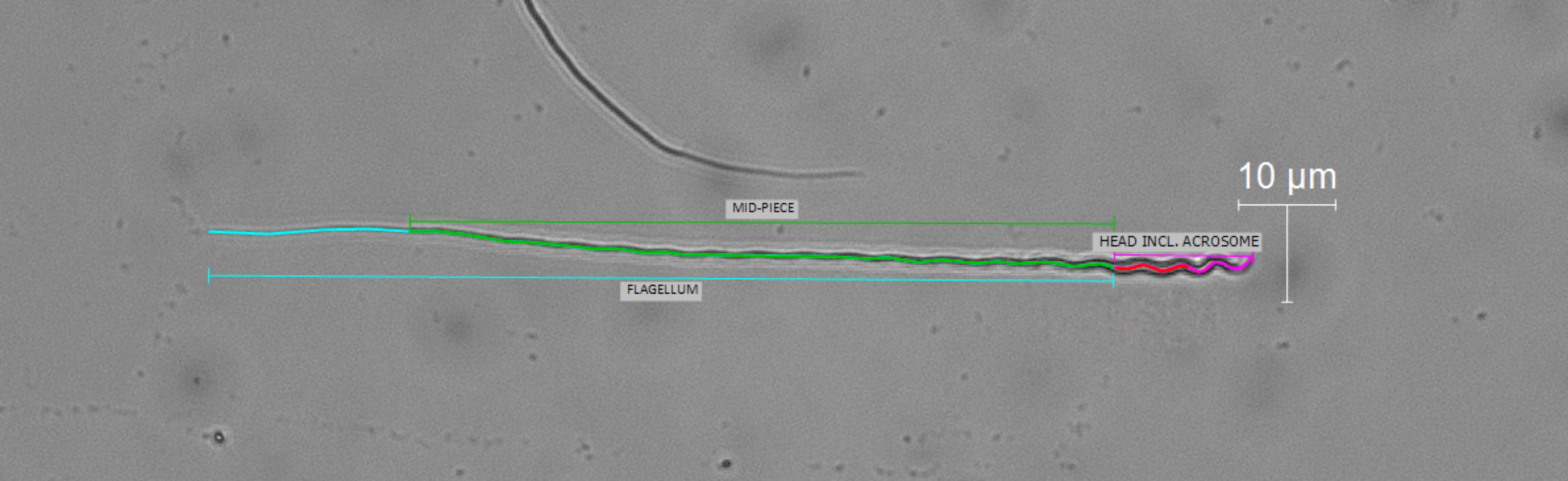
Length measurements of house sparrow sperm. Example of measurements of single sperm components: head: nucleus (red) and acrosome (pink), midpiece (green), and flagellum: tail (cyan) and midpiece (green). Total length was calculated by using the sum of flagellum and head length. Note that whereas the transition from midpiece to head and the end of the acrosome are visible in light microscopy; the acrosome and nucleus cannot be precisely differentiated and are portrayed here only to demonstrate the composite nature of the head measurement.

We prepared and measured sperm, as it is common practice in studies of avian sperm morphometry by using unstained sperm, bright field microscopy and formalin as a fixative (e.g. [17,21,23,25,42]). Specifically, we prepared 10μl aliquots onto microscope slides from the formalin-fixed samples. We only used slides that held a minimum of 100 sperm to account for potential sperm abnormalities [28] and photographed the first ten sperm that were intact and normal, i.e. that did not deviate in form from typical passerine sperm [44] such as e.g. deformations or loss of sperm components (see S1 Appendix in the supporting information). We only used sperm that were not covered by other sperm or detritus and always started in the upper left corner of a slide to avoid observer bias and to ensure that no sperm was mistakenly measured twice. Digital images of single sperm were taken with a Leica DFC450-C camera, mounted on a Zeiss Axioplan-2 microscope at x400 magnification using bright field settings. From these digital pictures, we then took three consecutive measurements to the nearest 0.01μm of the following three sperm traits: head (i.e. nucleus including acrosome), midpiece, and flagellum. We used the mean of the three consecutive measurements for statistical analyses. All measurements were taken by one observer only (GC) with the Leica Application Suite (LAS) software v4.2 using the LAS segment tool and centring the line within sperm and segmenting where necessary to follow the helical twists and natural curvature (Fig 4).

Total sperm length was calculated as the sum of the length of the flagellum and head because the calculated measurement correlated strongly with measured total sperm length (Pearson *r* = 0.92, df = 98, p<0.0001, *n* = 100 randomly chosen sperm using the function ‘runif()’ in R version 3.3.1 [45]).

Whereas massage and female dummy samples were indistinguishable under the microscope, on occasion, we could discriminate a faecal from a massage sample, if detritus was present in faecal samples. Throughout the measuring process, however, the observer was blind in respect to the question at test. To test how many sperm were needed to get a sufficiently precise estimate of individual sperm length, we initially measured 20 sperm per male, considering individual mean trait estimates using R^2^ of linear regression. Using the built-in function ‘sample()’ in R version 3.3.1 [45], we selected one of the three sperm components (i.e. midpiece) measured from 45 males and regressed means from measuring 5, 10, 15 midpieces against the "full" mean trait estimate when measuring 20 midpieces. The adjusted R^2^ can then be used to interpret how much of the variance, when measuring 20 sperm, is explained by each single predictor [46]. The adjusted R^2^ was 0.71, when using the mean length of 5 sperm per male, compared to 0.91 and 0.97 for 10 and 15 sperm, respectively. We thus concluded that measuring 10 sperm per male was sufficient to estimate individual sperm length. To establish observer repeatability, we randomly selected one microscope slide using the built-in function ‘sample()’ in R version 3.3.1 [45] and measured 20 sperm twice, leaving 48 hours between measurements to ensure independence of measurements.

### Statistical analyses

We fitted linear mixed models with Gaussian errors using the function ‘lmer’ from the package ‘lme4’ [47] in R version 3.3.1 [45] with the total length of single sperm components as respective response variables. We used the raw data from all sperm measured per male for linear mixed models (range 10 − 20 sperm per male) instead of using the mean or median of all sperm measured per male. Collection technique was fitted as a predictor variable (two levels: faecal and massage sampling). Because we did not obtain all samples from all males as anticipated, we excluded female dummy samples from analyses and added the relative order of the faecal to massage collection technique (i.e. first, second) as a fixed effect to the model. This choice of order is sensible, because sperm supplies are replenished in house sparrows over night [39]. Inbreeding depression can affect sperm morphology [48], and individual standardised multilocus heterozygosity (i.e. sMLH, calculated with the R package ‘inbreedR’ [49]) was therefore used as a proxy for inbreeding depression from marker data [49]. However, sMLH was not associated with the length of sperm traits (results not shown), which means that our findings were not affected by inbreeding depression, if at all present in our population, and we therefore did not keep this variable in the model. We added male ID, sample ID and cohort (i.e. year of birth) as random effects on the intercept to account for repeated measurements of individuals, non-independence of sperm measurements within experimental ejaculates, and potential cohort effects. Model fit and assumptions were validated by visual inspection of residuals [46]. Observer repeatability and individual male repeatability for length measurements were calculated with the R package ‘rptR’ [50] suitable for Gaussian data using 1000 bootstrap and 1000 permutations. We used the function ‘sim’ from the package ‘arm’ to calculate posterior distributions (n = 1000 draws) with flat priors from our linear mixed models [51] and report posterior means and 95% Bayesian Credible Intervals (CrI). CrI not overlapping zero are interpreted as a Frequentist p-value of < 0.05, and thus as a statistically significant result [46]. Hence, CrIs can be used to test null-hypotheses but doing so should not exclude acknowledging that CrIs provide more valuable information than p-values (e.g. uncertainty in the parameter estimate, how close the model estimate is to zero) [46,52]. The data and the R script are available at the Open Science Framework (DOI 10.17605/OSF.IO/RYCMN).

## Results

### Efficacy of sperm collection techniques

We collected sperm using three common methods, which allowed us to compare their efficacy in obtaining samples and sperm. Of 52 males tested on two days, only three males copulated with the female dummy, and for only two of these males could we collect experimental ejaculates. In total, we had five experimental ejaculates from two males available from female dummy sampling (one male copulated twice with the female dummy during trial 1 and both males copulated with the female dummy during trial 1 and trial 2). Because of the limited sample size, the sperm length measures from the female dummy samples were thus only used for descriptive summary statistics (Table 1) but omitted from further statistical analyses. Faecal sampling proved to be more successful and samples were obtained in 99 of 104 trials. Five defaecation trials were unsuccessful, because the males did not defaecate within ten minutes. Similarly successful as with faeces collection, massage failed only in five out of 104 trials and in all these cases, failure coincided with males exhibiting small cloacal protuberances [41], indicating low breeding condition [53] compared to the other experimental males. All five female dummy samples, 67 out of 99 faecal samples, and 71 out of 99 massage samples (6% more compared to faeces) could be used for sperm length measurements.

**Table 1:** Descriptive summary statistics of length measurements of house sparrow sperm.

	female dummy	massage samples	faecal samples
**total length**			
mean ± SD (μm)	98.70 ± 1.89	99.62 ± 2.40	99.21 ± 2.42
CV	1.91	2.41	2.44
range (μm)	96.23 − 100.62	93.44 −105.89	93.05 − 104.95
**head**			
mean ± SD (μm)	13.69 ± 0.45	13.80 ± 0.70	13.47 ± 0.78
CV	5.51	5.07	5.76
range (μm)	12.95 − 14.02	12.04 − 15.96	12.12 − 15.92
**midpiece**			
mean ± SD (μm)	68.85 ± 0.97	67.72 ± 1.67	67.50 ± 1.71
CV	1.41	2.46	2.54
range (μm)	67.76 − 70.37	64.04 − 71.02	62.83 − 70.89
**flagellum**			
mean ± SD (μm)	85.01 ± 1.56	85.83 ± 2.44	85.74 ± 2.30
CV	1.84	2.84	2.68
range (μm)	82.67 − 86.60	79.48 − 91.23	80.54 − 90.43

Summaries are based on 5 female dummy (*n* = 2 males), 71 massage (*n* = 45 males) and 67 faecal samples (*n* = 41 males) using averages of all sperm measured per male within collection technique treatment. CV: coefficient of variation calculated as 100*(sd/mean). SD: standard deviation.

### Sperm morphometrics

All sperm traits showed high observer measurement repeatability (>80%, Table 2) and repeatability within-males across days and methods (Table 2). Descriptive summary statistics (Table 2) demonstrate that our length measurements are representative of the species because they are similar to published records (e.g. [24,44,54]).

**Table 2:** (a) Observer repeatability, and (b) individual male repeatability for house sparrow sperm length measurements.

	*n* measures	repeatability	CI	*P*
**a) observer repeatability**				
total length	20	1.00	(0.99, 1.00)	<0.001
head	20	0.95	(0.89, 0.98)	<0.001
midpiece	20	0.83	(0.63, 0.93)	<0.001
flagellum	20	0.98	(0.94, 1.00)	<0.001
**b) male repeatability**				
total length	43	0.80	(0.69, 0.87)	<0.001
head	43	0.61	(0.43, 0.74)	<0.001
midpiece	43	0.77	(0.64, 0.85)	<0.001
flagellum	43	0.81	(0.70, 0.88)	<0.001

Confidence intervals (CI) and p-values (*P*) were obtained from using 1000 bootstrap and 1000 permutation tests [50].

**Fig 5:**
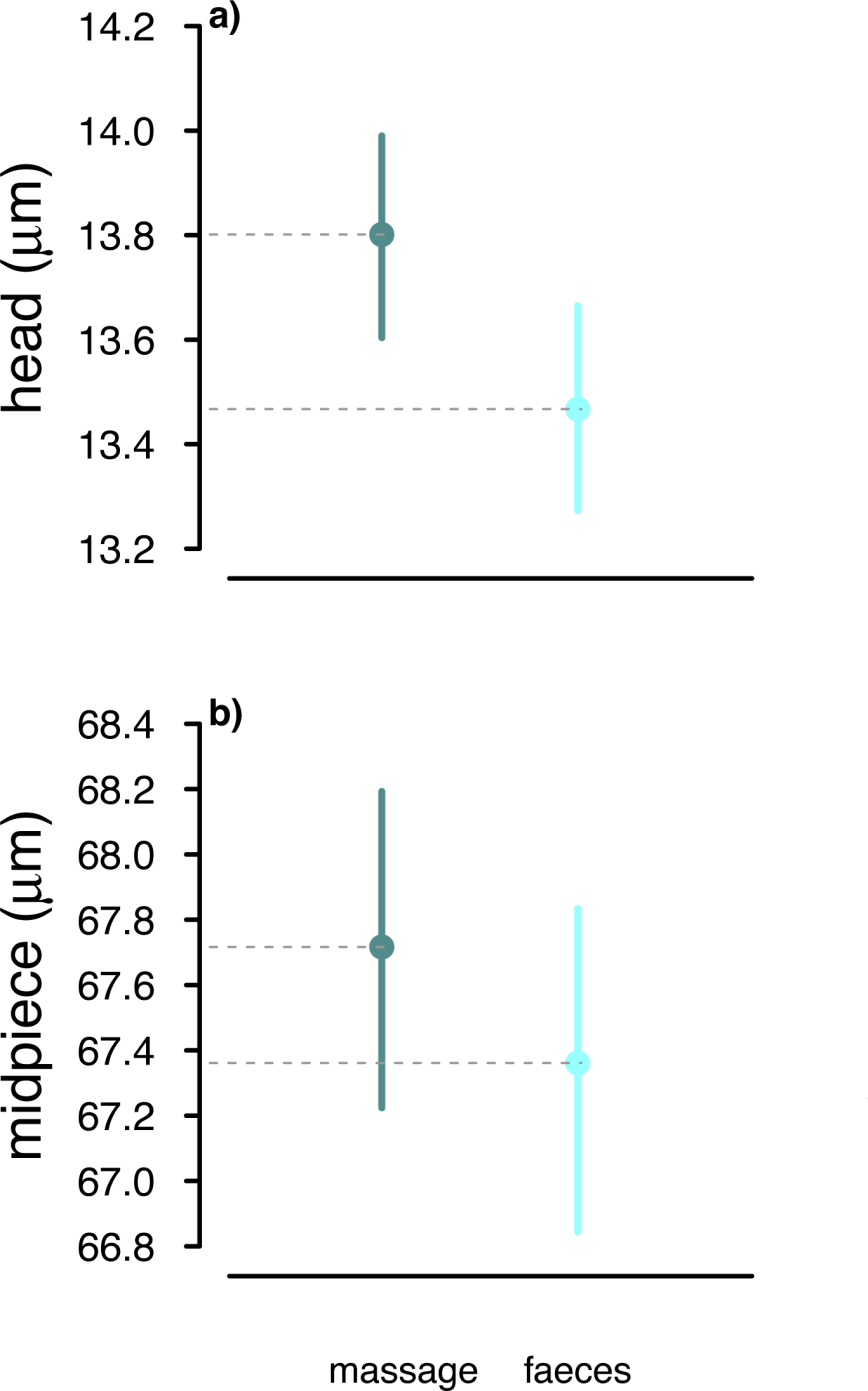
Sperm length (μm) differences in relation to sperm sampling method in house sparrows controlling for relative order of massage to faecal sampling. Sperm heads (a), and midpieces (b) were significantly shorter sampled from faeces [17] (823 sperm from 41 males) compared to abdominal massage samples [19,20] (822 sperm from 45 males). Filled dots represent means and vertical lines represent 95% Bayesian Credible Intervals (CrI).

**Fig 6:**
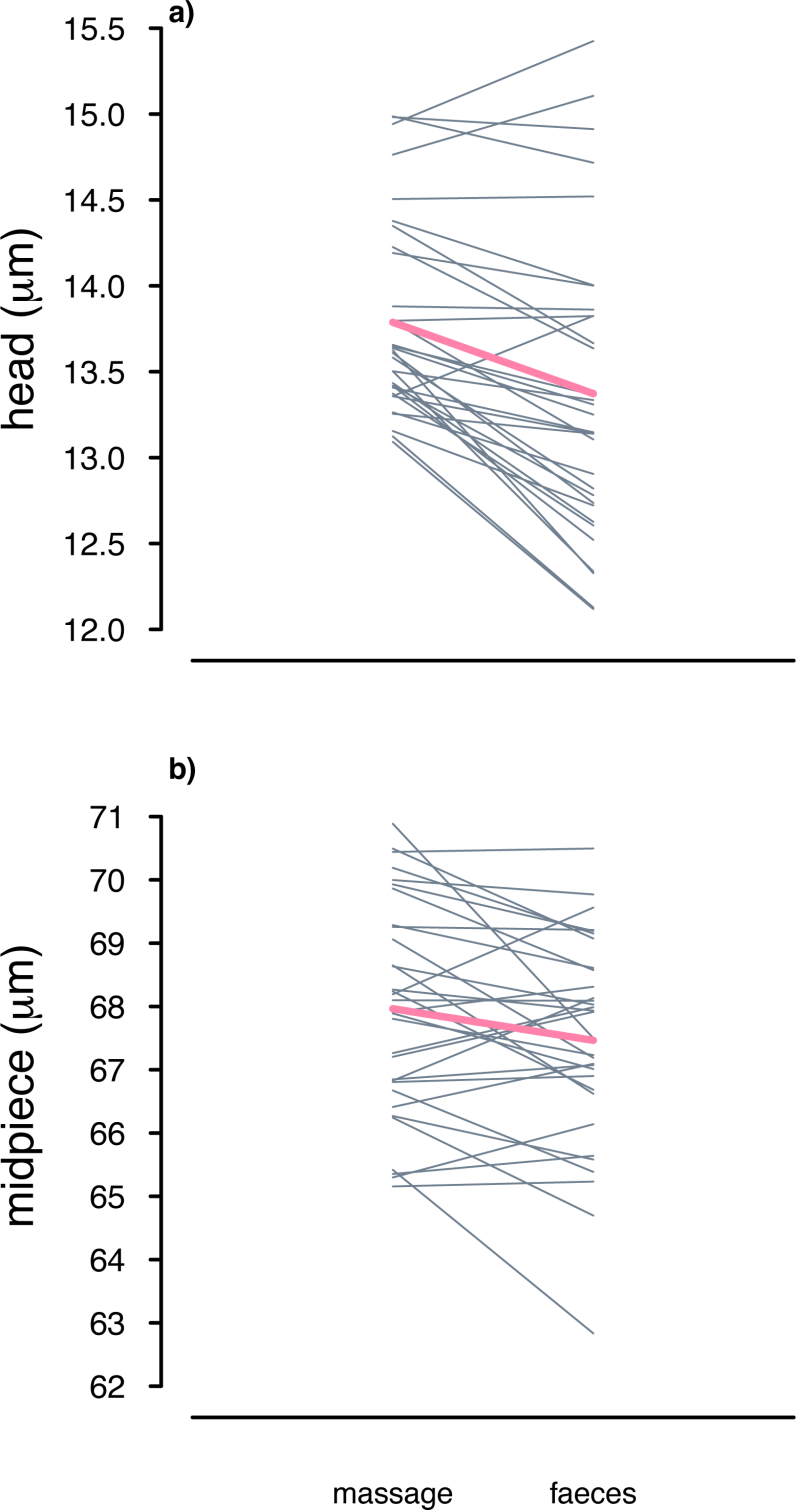
Individual males' sperm length (μm) differences in relation to sperm sampling method in house sparrows. We present individual raw data of males (*n* = 16 males) for which we had a total of two faecal and two massage samples. Thin grey lines connect the measurements for massage and faecal samples of individual males per trial. Pink lines connect the average measurements of massage and faecal sampled sperm across trials.

Our analysis of collection method affecting sperm length controlling for relative order of sampling showed that, on average, the total length of faecal sampled sperm across males was 0.41 μm less than of sperm collected by massage (Table 3). This difference, albeit small, was statistically significant, and explained by shorter sperm heads and midpieces in faecal compared to massage samples (Table 3, Fig 5). Sperm flagella did not differ in length between methods (Table 3). Furthermore, because we ultimately compared only two methods for sperm length differences, due to the female dummy experiment resulting in small sample sizes, a paired *t*-test could be seen as a *post-hoc* statistical alternative to linear mixed models. In contrast to the linear mixed models, only males with massage and faecal samples obtained during both trials were used for the paired *t*-test (*n* = 16 instead of 47 males), so relative order of methods, inbreeding etc. did not need to be controlled for. Using this simpler test confirmed our result of shorter sperm heads (paired *t*-test *t*_*31*_ *=* 5.26, *P* < 0.001) and midpieces (paired *t*-test *t*_*31*_ *=* 2.55, *P* = 0.02), but not flagella (paired *t*-test *t*_*31*_ *=* 0.27, *P* = 0.79) in faeces compared to massage samples (Fig 6). Hence, despite small effect sizes, the results remained robust when using a smaller dataset.

**Table 3:** Length (μm) of house sparrow sperm in relation to sperm sampling method controlling for relative order of faecal [17] to massage samples [19,20].

	total length	head	midpiece	flagellum
(intercept)	99.46	13.83	67.57	85.64
	(98.74, 100.22)	(13.60, 14.06)	(67.07, 68.06)	(84.93, 86.36)
method (faeces)	**-0.41**	**-0.34**	**-0.35**	-0.08
	**(-0.78, -0.04)**	**(-0.49, -0.16)**	**(-0.61, -0.08)**	(-0.40, 0.25)
order (second)	-0.03	-0.04	0.28	-0.01
	(-0.40, 0.34)	(-0.22, 0.13)	(-0.04, 0.54)	(-0.33, 0.31)
Random effects				
male ID	7.80	0.36	4.19	8.93
	(5.34, 10.89)	(0.26, 0.48)	(2.79, 5.96)	(6.14, 12.25)
cohort	0	0	0	0
	(0, 0)	(0, 0)	(0, 0)	(0, 0)
sample ID	0.28	0.16	0.03	0.06
	(0.21, 0.35)	(0.12, 0.19)	(0.03, 0.04)	(0.04, 0.07)
residual variance	3.03	0.87	2.61	2.97
	(2.93, 3.15)	(0.84, 0.89)	(2.53, 2.70)	(2.87, 3.08)

We present posterior means and CrI for each linear mixed model based on 1645 sperm from 47 males. Meaningful CrI not overlapping zero are in bold.

A follow-up analysis of a subset of 13 males also showed no quantitative difference of deformed sperm in faecal compared to massage sampled sperm (mean number deformed sperm among 100 sperm ± SD: faecal: 8.9 ± 8.04; massaged: 13.46 ± 10.34, paired Wilcoxon signed rank test, *V* = 20, *P* = 0.15, see also S1 Appendix in the supporting information for more details).

## Discussion

In contrast to the assumption that sperm morphometry does not differ between collection methods (e.g. [55]), we found that sperm were shorter when collected from faecal instead of massage samples. Specifically, we found heads and midpieces in faecal samples to be shorter compared to massage samples, while no difference was found in flagella length.

Sperm length differences can have methodological or biological origins. For instance, sperm are sensitive to handling and storage [56] and sperm deformation can occur because of sample preparation [57,58]. However, sperm deformation cannot explain our results, because we only measured intact, non-deformed sperm. Also, handling and storage cannot explain our finding either, as we collected samples within a short period of time, sample type was randomised within sampling event, and samples were prepared and measured as one batch. Additionally, we adopted published procedures for all three methods, used fixatives of identical concentrations and neither PBS nor formalin are known to affect avian sperm morphology [59]. Instead, biological factors such as the intensity of sperm competition [60], genome size [28] or sperm maturation [61] can affect sperm size. Here, we had repeated measures of individual males in identical environments, which remove the possibility for intrinsic effects to account for our results. Specifically, our additional analysis of using a paired *t*-test confirmed our results from linear mixed models and was restricted to males for which we collected faecal and massage samples during both events, so here males served as their own control. Therefore, the observed sperm size differences in heads and midpieces between methods might be explained by sperm in faeces resembling a different subpopulation of sperm within males and could be indicative of differences in the degree of post-meiotic sperm maturation [62,63].

Highlighting sperm maturation can help interpreting our result of differences in sperm length between methods. Sperm production takes place in the seminiferous tubules of the testis where primordial germ cells transform into spermatids [64]. Spermatids then undergo further development to mature into fully functioning spermatozoa. One feature of this maturation process called spermiogenesis is the elongation of sperm heads and flagella [61,64]. In other words, fully matured sperm are longer compared to less mature sperm. Furthermore, passerine spermiogenesis can be categorised according to work done at the ultrastructural level on house sparrow sperm [61]. Importantly, sperm at the last two stages of the maturation process, which are called stage 5 and 6 [61], resemble morphologically typical passerine sperm [44], so sperm from stage 5 onwards would have qualified to be measured in our study. However, in regard to sperm length, stage 5 sperm differ from stage 6 sperm in that the heads and midpieces are not fully elongated [44,61]. It is thus possible that the observed difference in sperm length is explained by immature sperm, (i.e. stage 5 ≤ sperm < stage 6 [61]), being defaecated rather than stored for copulation. An alternative explanation is that both faecal and massage sampled sperm have finished spermiogenesis, and thus the elongation of sperm components, but it is more senescent sperm *sensu* [31] that are defaecated instead of being stored. Indeed, the continuous release of sperm in reproductively active males has been speculated to remove excessive or senescent sperm [65], which would ensure that high-quality sperm are available for insemination. The length difference that we found could thus be explained, in principle, by senescent sperm in faeces versus fresh sperm in the seminal glomera. However, the literature does not suggest changes in sperm length as an accompanying feature of post-meiotic sperm senescence [31], which makes this explanation less likely. Moreover, it is unclear which mechanisms might account for either the idea of selective sperm loss of senescent [18] or less mature sperm with defaecation. Under both scenarios, however, our results could be regarded as indirect evidence to support an adaptive explanation of sperm defaecation [65], assuming that sperm of lower fertilisation efficiency are defaecated. Another factor that might play a role is that sperm sampled from faeces might experience higher osmotic stress compared to sperm sampled from abdominal massage. Uric acid attributes little to osmotic pressure [15], but the osmotic concentration in faeces might still be higher than the osmotic concentration of epididymal secretions, which might lead to shrinkage of faecal sampled sperm. Whereas we can only speculate about the biological causes of our results, the important implication from our findings is that when collection techniques are mixed in avian sperm studies, the method of sperm sampling should be controlled for statistically to reduce uncertainty in statistical models [35,36].

To explain aspects of avian evolutionary biology through sperm biology it is desirable to collect natural ejaculates. Unarguably, the method that comes closest to sampling natural ejaculates is the stuffed female dummy technique [40], especially with its adaptation in fowl where a harness is fitted to live females [66]. The stuffed female dummy technique might work well in some species [67], but despite a peculiar anecdote that house sparrow males might require little stimuli to initiate copulation [68], it did not work well in our populations (we also tried the female dummy collection technique in the field on a wild population with no success (Girndt personal observation). Commonly, only a subset of males copulate with a female dummy [67]. In our experiment this subset was very small (6% of 52 males) and the behavioural difference between the three males that did copulate with the female dummy and the majority of males that did not, differed markedly: rapid and repeated copulation versus complete ignorance. Also, a pilot study on 45 reproductively active males in our population in 2014 (Beirer *et al* unpublished) tested the female dummy three times using a single-male set-up. There, a total of two males copulated with the female dummy, and the number of males that approached it within a vicinity of 20 centimetres decreased from 11 males during trial 1 to two males during the final, third trial. Thus, repeated exposure to the female dummy seemed to counteract the little initial interest she had sparked. Therefore, we doubt that house sparrows could be trained to copulate with the female dummy, but we cannot exclude that a more sophisticated female dummy, e.g. a copulation robot mimicking female solicitation [43] might be more successful as a sperm collection device in house sparrows. The massage method yielded more samples than the female dummy method, and both methods have additional advantages and disadvantages that are worth highlighting. Whereas little to no training will be required to collect the wet part of a male's faeces, massaging passerines demands practice and training. Also, mostly clean samples are obtained from massage, whereas faecal samples hold detritus that can obstruct viewing sperm under the microscope. Lastly, faecal sperm sampling has been advertised as less invasive [17]. Under the original medical interpretation of the word: no introduction of instruments into the body [69], neither the faecal, nor the female dummy or massage technique are invasive. Using a more applied interpretation of "non-invasive" as minimised handling stress, we argue that faecal sperm sampling is only non-invasive in the unlikely scenario that a researcher finds a fresh [17], pipettable bird's faeces in the field, and can assign it to a species/individual without restraining it. This scenario immediately limits the questions that can be answered by it (e.g. describing gross sperm morphology in a new species). Instead, a literature search of studies citing [17] suggested that faecal sperm sampling was commonly applied by following the sampling method described in [17], which involved handling and constraining males. Handling males is also needed for sperm collection via massage but from our experience massaging males requires less time than faecal sperm sampling.

To the best of our knowledge, our study is the first that experimentally tested whether the faecal method can be used interchangeably with massage or the female dummy technique, using a randomised experimental design with repeated measures from individual male house sparrows. We found a statistically significant, albeit small, difference between sperm length of massage and faecal sampled sperm that could resemble length differences between sperm at potentially different maturational stages. Importantly, our effect size of mean head differences for instance, is similar to effect sizes described in bird, mammal or fish sperm literature (e.g. [65,70–73]). Consequently, if sperm length varies within males according to method (see also [29,74] highlighting qualitative sperm differences), earlier results that did not take methodological differences into account might have made an interpretation of results more difficult. We encourage other scientists working in avian sperm biology to replicate our approach to test its generality in other species. In addition, where collection of natural ejaculates is improbable, we recommend the abdominal massage over the faecal sampling technique due to our findings, its advantage of giving cleaner samples and no difference in invasiveness.

## Supporting information

S1 Movie House sparrow male copulating with a female dummy. This video gives an example of a male house sparrow, *Passer domesticus*, copulating with a stuffed female dummy house sparrow. The female dummy had lived in the population for seven years before she died of a natural cause in 2014. The video was taken to illustrate an experimental copulation with the female dummy. It was not part of the experiment described in the manuscript.
(MP4)

S1 Appendix Sperm abnormality procedures. Description of methods.
(DOCX)
