# Supplementary material for: Method matters: Experimental evidence for shorter avian sperm in faecal compared to abdominal massage samples
Source: PLoS One. 2017 Aug 16;12(8):e0182853. doi: 10.1371/journal.pone.0182853 (PMC5559096; doi:10.1371/journal.pone.0182853)
Supplement: S1 Appendix — Description of methods. (DOCX) [file pone.0182853.s002.docx]

**S2 Text. Sperm abnormality procedures.** Sperm were scored for abnormalities in a subset of 13 house sparrow, *Passer domesticus*, males in both their faecal and massage sampled sperm. Sperm were classified as abnormal if they deviated from the typical passerine's (oscine) shape*,* which consists of an acrosome with a helical membrane, a nucleus, also of helical shape, and the flagellum consisting of the midpiece whose mitochondria form a helix around the axoneme and the non-helical tail [1,2]. Abnormalities were scored for sperm heads, midpieces and tails and could consist, for instance, of heads bent for more than 90°, bulbous or completely linear heads, pinheads, or absence of helical twists, as well as cytoplasmic droplets. Midpiece deformities included distal cytoplasmic droplets and bends of more than 90°. Sperm tails were scored as abnormal if they appeared coiled, stubbed, had cytoplasmic droplets or occurred in aberrant numbers. The protocol for assessing the proportion of abnormal to normal sperm within experimental ejaculates was as such that we scored the first 100 unobstructed sperm using a 400 magnification and the Zeiss Axioplan-2 microscope. Again, we always started in the left upper corner of the microscope slide to avoid observer bias and duplicated scoring. The observer scoring sperm (AG) was blind in regard to the question at test.

**References**

1. Jamieson B. Avian spermatozoa: structure and phylogeny. In: Jamieson B,

editor. Reproductive Biology and Phylogeny of Birds. Jersey: Science Publishers; 2007. pp. 349–398.

2. Ballowitz E. Untersuchungen über die Struktur der Spermatozöen, zugleich ein Beitrag zur Lehre vom feineren Bau der contraktilen Elemente. Theil I. Die Spermatozoen der Vögel. Arch f mikroskop Anat. 1888;Bd.32.
